# Anti-*Porphyromonas gingivalis* lipopolysaccharide antibody in rheumatoid arthritis patients with emphysema

**DOI:** 10.3389/fmed.2025.1654271

**Published:** 2025-09-22

**Authors:** Shomi Oka, Takashi Higuchi, Kota Shimada, Misuzu Fujimori, Atsushi Hashimoto, Akiko Komiya, Koichiro Saisho, Norie Yoshikawa, Michita Suzuki, Toshihiro Matsui, Naoshi Fukui, Kiyoshi Migita, Shigeto Tohma, Hiroshi Furukawa

**Affiliations:** ^1^Department of Rheumatology, NHO Tokyo National Hospital, Kiyose, Japan; ^2^Clinical Research Center for Allergy and Rheumatology, NHO Sagamihara National Hospital, Sagamihara, Japan; ^3^Department of Rheumatology, NHO Sagamihara National Hospital, Sagamihara, Japan; ^4^Department of Rheumatic Diseases, Tokyo Metropolitan Tama Medical Center, Fuchu, Japan; ^5^Department of Rheumatology, NHO Himeji Medical Center, Himeji, Japan; ^6^Department of Internal Medicine, Sagami Seikyou Ganka Naika, Sagamihara, Japan; ^7^Department of Clinical Laboratory, NHO Sagamihara National Hospital, Sagamihara, Japan; ^8^Department of Orthopedics/Rheumatology, NHO Miyakonojo Medical Center, Miyakonojo, Japan; ^9^Tanimura Hospital, Nobeoka, Japan; ^10^Department of Internal Medicine, NHO Nagoya Medical Center, Nagoya, Japan; ^11^Department of Life Sciences, Graduate School of Arts and Sciences, The University of Tokyo, Tokyo, Japan; ^12^Clinical Research Center, NHO Nagasaki Medical Center, Omura, Japan; ^13^Department of Gastroenterology and Rheumatology, Fukushima Medical University School of Medicine, Fukushima, Japan; ^14^Department of Internal Medicine, St. Francis Hospital, Nagasaki, Japan

**Keywords:** rheumatoid arthritis, anti-*Porphyromonas gingivalis* antibodies, emphysema, chronic lung disease, citrullinated peptides

## Abstract

**Objective:**

Lung diseases that are chronic (emphysema [EMP], airway disease, interstitial lung disease) can complicate rheumatoid arthritis (RA) as extra-articular manifestations. Anti-*Porphyromonas gingivalis* (*P. gingivalis*) antibody (Ab) has been analyzed for the diagnosis of periodontal disease and RA patients showed increased anti-*P. gingivalis* Ab levels. However, amounts of anti-*P. gingivalis* Ab in RA complicated with chronic lung disease (CLD) are unknown. We measured anti-*P. gingivalis* Ab in cases of RA with CLD.

**Methods:**

Anti-*P. gingivalis* lipopolysaccharide (LPS) Ab were measured in RA patient sera by enzyme immunoassay.

**Results:**

Anti-*P. gingivalis* LPS Ab levels in RA with EMP were significantly lower than in RA cases without CLD (*P* = 0.0190, mean ± standard deviation [SD], 32.7 ± 64.2 [×10^3^U/mL] vs. 403.5 ± 2552.7 [×10^3^U/mL]). Multiple logistic regression analyses revealed the independence of this association. Anti-*P. gingivalis* LPS Ab amounts were increased in RA without CLD (*P* = 0.0412) compared with healthy controls (77.6 ± 183.7 [×10^3^U/mL]).

**Conclusion:**

Anti-*P. gingivalis* LPS Ab levels in RA with EMP were lower compared with RA without CLD. Citrullinated peptides were mainly generated in the lungs of RA with EMP and the oral cavity of RA without CLD, suggesting the heterogeneity of RA.

## Introduction

Pathogenic factors of the chronic autoimmune disease rheumatoid arthritis (RA) featuring destruction of synovial joints (1), are poorly understood. Chronic lung diseases (CLD) (emphysema [EMP], airway disease [AD], interstitial lung disease [ILD]) (2), can frequently complicate RA, which results in a poor prognosis (3–8).

Rheumatoid arthritis patients generate many autoreactive antibodies, such as rheumatoid factor (RF) and anti-citrullinated peptide antibody (ACPA), which are thought to be pathogenic. Citrullinated peptides generated in lungs under the influence of smoking, lead to the production of ACPA. Smoking also confers a poor prognosis in RA patients (9). Periodontal disease, associated with infection by *Porphyromonas gingivalis* (*P. gingivalis*) has links to RA (10). Endogenous peptidyl-arginine-deiminase from *P. gingivalis* mediates oral cavity citrullinated peptide production, leading to the generation of ACPA (1, 11).

Anti-*P. gingivalis* antibody (Ab) analysis for the diagnosis of periodontal disease (12) showed anti-*P. gingivalis* Ab titers were decreased after treatment (13, 14). The levels of anti-*P. gingivalis* whole-cell Ab highly correlated to anti-*P. gingivalis* lipopolysaccharide (LPS) Ab levels (15). Anti-*P. gingivalis* Ab levels are increased in atherosclerosis (16), hyperlipidemia (17), RA (10), and chronic kidney disease (18). However, treatment of periodontal disease did not alter RA disease activity (19, 20).

Serum anti-*P. gingivalis* Ab levels might reflect *P. gingivalis* infection, which increases the risk of developing some diseases. The amounts of anti-*P. gingivalis* LPS Ab in RA complicated with CLD could be lower than those without CLD, since citrullinated peptides might be generated in lung of RA with CLD and in oral cavity of RA without CLD, respectively. However, there are few reports on amounts of anti-*P. gingivalis* Ab in RA complicated by CLD. This investigation measured anti-*P. gingivalis* Ab in RA complicated by CLD.

## Material and methods

### Patients

Overall, 637 RA patients with chest computed tomography findings and 52 healthy controls (HCs) at Miyakonojo Medical Center, Himeji Medical Center, Nagasaki Medical Center, Tokyo National Hospital, Nagoya Medical Center, and Sagamihara National Hospital were recruited. All RA cases fulfilled the RA criteria (21, 22). Those with ILD, AD, EMP, or no CLD [CLD(−)] were diagnosed using chest computed tomography as described elsewhere (23–25): ILD (irregular linear opacities and honeycombing, bilateral ground-glass attenuation patterns predominantly in subpleural and basal regions), AD (centrilobular or peribronchial nodules and branching linear structures, bronchial dilatation, bronchial wall thickening, or atelectasis), EMP (low attenuation area or bullae), or CLD(−) (no abnormalities in computed tomography images). RA patients with the other chest computed tomography patterns were excluded from this study. Steinbrocker stages were evaluated as described (26).

This study complied with the Declaration of Helsinki. The study received approval from the Research Ethics Committee of Tokyo National Hospital (190010) and Research Ethics Committees of the institutes involved. Informed written consent was attained from all participants.

### Detection of serum anti-P. gingivalis LPS Ab

Serum anti-*P. gingivalis* LPS Ab was measured by a Human anti-P. gingivalis LPS Ab ELISA kit (Chondrex, Inc., Woodinville, WA, USA), as instructed by the manufacturer (27–29).

### Statistical analysis

Rheumatoid arthritis patient clinical characteristics were compared using the Student *t*-test or Fisher’s exact test with the use of a 2 × 2 contingency table. Anti-*P. gingivalis* LPS Ab amounts were compared by Student’s *t*-test or Mann–Whitney *U*-test. Correlations between anti-*P. gingivalis* LPS Ab amounts, ACPA titers, and disease activity score 28 (DAS28) were evaluated with Pearson correlation coefficient values. Independent associations of amounts of anti-*P. gingivalis* LPS Ab with EMP in RA were evaluated by multiple logistic regression. The associations of amounts of anti-*P. gingivalis* LPS Ab with EMP were evaluated when conditioned on each clinical manifestation. The associations of each clinical manifestation with EMP were evaluated when conditioned on amounts of anti-*P. gingivalis* LPS Ab.

## Results

### Clinical characteristics of RA patient subsets

Comparisons of RA subset clinical characteristics were compared to those with RA cases without CLD (Table 1). Mean ages, male percentages, onset age, ever smoker percentage, and percentage of Steinbrocker stages III or IV, were higher in RA complicated with ILD or EMP. The body mass index (BMI) was lower and the age at onset as well as the mean age were higher for RA with AD. Increased RF levels were observed for RA with ILD, and ACPA levels were decreased in RA with EMP.

**TABLE 1:** Clinical characteristics of RA subsets.

	ILD		AD		EMP		CLD(−)
		*P*		*P*		*P*	
Number	167		167		39		264
Age, years (SD)	69.1 (8.7)	2.35 × 10^–14^	68.0 (10.5)	1.81 × 10^–9^	66.8 (8.2)	0.0005	61.3 (11.8)
Male, *n* (%)	53 (31.7)	0.0016	28 (16.8)	0.7955	24 (61.5)	5.50 × 10^–8^	48 (18.2)
Age at onset, years (SD)	56.7 (14.4)	8.19 × 10^–10^	54.2 (15.6)	1.64 × 10^–5^	57.4 (11.7)	1.48 × 10^–5^	47.7 (14.2)
Steinbrocker stage III and IV, n (%)	68 (41.7)	0.0098	84 (52.8)	0.7628	13 (33.3)	0.0158	145 (54.7)
Ever smoker, *n* (%)	71 (45.8)	0.0081	55 (37.2)	0.3262	30 (85.7)	1.57 × 10^–9^	80 (32.3)
BMI, kg/m^–2^, (SD)	22.9 (4.0)	0.0138	20.8 (3.7)	0.0064	21.2 (3.2)	0.2242	21.8 (3.4)
RF, IU/ml (SD)	501.8 (1136.2)	0.0071	366.4 (899.5)	0.3303	285.6 (527.3)	0.0693	361.0 (1079.4)
ACPA, U/ml (SD)	345.4 (714.3)	0.3345	342.2 (382.4)	0.5559	203.1 (251.2)	0.0382	295.2 (318.2)
DAS28	3.0 (1.1)	0.3073	3.2 (1.2)	0.0254	2.9 (0.9)	0.8735	2.9 (1.1)

RA, rheumatoid arthritis; ILD, interstitial lung disease; AD, airway disease; EMP, emphysema; CLD, chronic lung disease; BMI, body mass index; RF, rheumatoid factor; ACPA, anti-citrullinated peptide antibody; SD, standard deviation; Ab, antibody; DAS, disease activity score. The average or positive number in each group is shown. Phenotype frequencies or SDs are shown in parenthesis. Differences compared with the CLD(−) population were tested by Fisher’s exact test using 2 × 2 contingency tables or Student’s *t*-test.

### Detection of anti-P. gingivalis LPS Ab in RA

**FIGURE 1:**
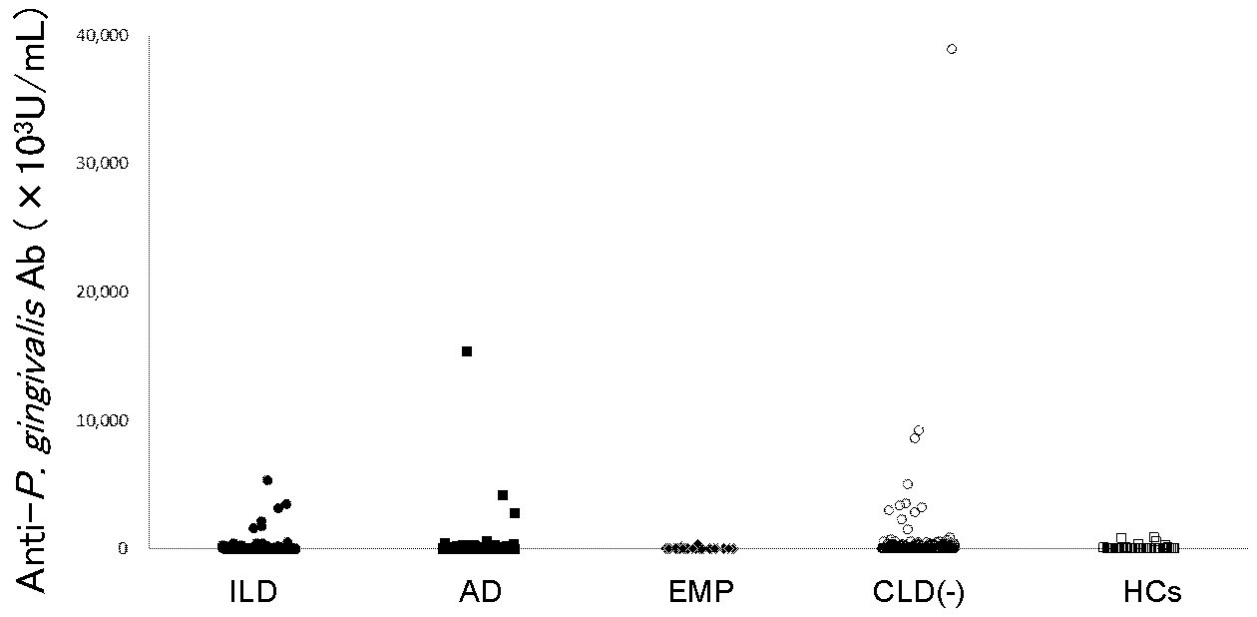
Anti*-P. gingivalis* LPS antibody levels in RA patients and controls. Anti-*P. gingivalis* LPS antibody levels are shown. Filled circles, filled squares, filled diamonds, empty circles, and empty squares represent RA with ILD, RA with AD, RA with EMP, RA without CLD, and HCs, respectively. RA: rheumatoid arthritis, ILD: interstitial lung disease, AD: airway disease, EMP: emphysema, CLD: chronic lung disease, HCs: healthy controls, LPS: lipopolysaccharide.

Anti-*P. gingivalis* LPS Ab was detected in RA patient sera (Figure 1) and comparisons of titers of each RA subset with those without CLD were performed (Table 2). Anti-*P. gingivalis* LPS Ab levels in RA with EMP (*P* = 0.0190, mean ± standard deviation [SD], 32.7 ± 64.2 [×10^3^U/mL]) were significantly lower than those in CLD(−)RA (403.5 ± 2552.7 [×10^3^U/mL]).

**TABLE 2:** Association of anti-*P. gingivalis* LPS Ab with the RA subsets.

		*t*-test	*U*-test
	Anti-*P.gingivalis* Ab, X10^3^U/mL (SD)	*P*	*P*
ILD	157.5 (597.7)	0.1333	0.0009
AD	186.1 (1248.2)	0.2386	0.0002
EMP	32.7 (64.2)	0.0190	0.0005
CLD(+)	157.3 (925.8)	0.1342	3.73 × 10^–6^
CLD(-)	403.5 (2552.7)		

RA, rheumatoid arthritis; ILD, interstitial lung disease; AD, airway disease; EMP, emphysema; CLD, chronic lung disease; SD, standard deviation; LPS, lipopolysaccharide; Ab, antibody. The mean of each group is shown. SDs are shown in parentheses. Differences compared with the CLD(−) population were tested with the Student’s *t*-test or Mann–Whitney *U*-test.

The correlation of anti-*P. gingivalis* LPS Ab titers, ACPA levels, and DAS28 were showed in Supplementary Table 1. The weak correlation of anti-*P. gingivalis* LPS Ab amounts and DAS28 was detected in the RA patients (correlation coefficient 0.09, 95% confidence interval [CI] 0.00–0.17, *P* = 0.0470), especially in CLD(−)RA (correlation coefficient 0.14, 95% CI 0.02–0.26, *P* = 0.0225).

The effects of smoking on anti-*P. gingivalis* LPS Ab titers were analyzed and the results were shown in Supplementary Table 2. Anti-*P. gingivalis* LPS Ab titers were not different between ever and never smoker groups, though anti-*P. gingivalis* LPS Ab titers in RA with EMP (*P* = 0.0003, mean ± SD, 18.9 ± 16.4 [×10^3^U/mL]) were significantly lower than CLD(−)RA (240.2 ± 1088.6 [×10^3^U/mL]) in never smoker group. The effects of smoking on ACPA titers were also analyzed (Supplementary Table 3), and no association was detected in this subgroup analysis.

### Anti-P. gingivalis LPS Ab is independently related to EMP in RA

Multiple logistic regression analysis was performed to omit the impact of patient clinical characteristics on anti-*P. gingivalis* LPS Ab linked to EMP in RA (Table 3). Univariate analysis demonstrated anti-*P. gingivalis* LPS Ab was significantly linked to EMP (*P* = 0.0123, odds ratio [OR] 0.99, 95% CI 0.98–1.00) and this persisted to be significant when conditioned on age (*P*_*adjusted*_ = 0.0172, OR 0.99, 95% CI 0.99–1.00), sex (*P*_*adjusted*_ = 0.0243, OR 0.99, 95% CI 0.99–1.00), age at onset (*P*_*adjusted*_ = 0.0221, OR 0.99, 95% CI 0.99–1.00), Steinbrocker stage (*P*_*adjusted*_ = 0.0141, OR 0.99, 95% CI 0.98–1.00), smoking status (*P*_*adjusted*_ = 0.0231, OR 0.99, 95% CI 0.99–1.00), BMI (*P*_*adjusted*_ = 0.0135, OR 0.99, 95% CI 0.98–1.00), RF (*P*_*adjusted*_ = 0.0164, OR 0.99, 95% CI 0.98–1.00), or ACPA (*P*_*adjusted*_ = 0.0193, OR 0.99, 95% CI 0.99–1.00), respectively. Thus, these data suggested that anti-*P. gingivalis* LPS Ab was independently associated with EMP in RA.

**TABLE 3:** Multiple logistic regression analysis of anti-*P. gingivalis* LPS Ab and each clinical manifestation for RA with EMP.

	Unconditioned			OR and *P* values of anti-*P. gingivalis* LPS Ab when conditioned on each clinical manifestation			OR and *P* values of each clinical manifestation when conditioned on anti-*P. gingivalis* LPS Ab		
					
	OR	95%CI	*P*	OR_*adjusted*_	95%CI	*P*_*adjusted*_	OR_*adjusted*_	95%CI	*P*_*adjusted*_
Anti-*P. gingivalis* LPS Ab	0.99	(0.98–1.00)	0.0123	NA	NA	NA	NA	NA	NA
Age, years	1.05	(1.02–1.09)	0.0060	0.99	(0.99–1.00)	0.0172	1.05	(1.01–1.09)	0.0099
Male	7.15	(3.50–14.63)	7.12 × 10^–8^	0.99	(0.99–1.00)	0.0243	6.85	(3.28–14.27)	2.87 × 10^–7^
Age at onset, year	1.06	(1.03–1.09)	9.59 × 10^–5^	0.99	(0.99–1.00)	0.0221	1.05	(1.02–1.08)	0.0004
Steinbrocker stage	0.60	(0.45–0.82)	0.0011	0.99	(0.98–1.00)	0.0141	0.62	(0.45–0.85)	0.0028
Ever smoker	12.37	(4.63–33.04)	5.29 × 10^–7^	0.99	(0.99–1.00)	0.0231	12.15	(4.50–32.81)	8.45 × 10^–7^
BMI, kg/m^–2^	0.94	(0.84–1.04)	0.2186	0.99	(0.98–1.00)	0.0135	0.96	(0.86–1.07)	0.4371
RF (IU/ml)	1.00	(1.00–1.00)	0.0059	0.99	(0.98–1.00)	0.0164	1.00	(1.00–1.00)	0.0166
ACPA (U/ml)	1.00	(1.00–1.00)	0.0463	0.99	(0.99–1.00)	0.0193	1.00	(1.00–1.00)	0.0939

RA, rheumatoid arthritis; EMP, emphysema; OR, odds ratio; CI, confidence interval; BMI, body mass index; RF, rheumatoid factor; ACPA, anti-citrullinated peptide antibody; NA, not assessed; LPS, lipopolysaccharide; Ab, antibody. In the left column, *P*, OR, and 95%CI were calculated by univariate logistic regression analysis for RA with EMP. In the center column, *P*_*adjusted*_, and OR_*adjusted*_, and 95%CI of anti-*P. gingivalis* LPS antibody were calculated by multivariate logistic regression analysis for RA with EMP, when conditioned on each clinical manifestation (for example, anti-*P. gingivalis* antibody and age). In the right column, *P*_*adjusted*_, and OR_*adjusted*_, and 95%CI of each clinical manifestation were calculated by multivariate logistic regression analysis for RA with EMP, when conditioned on anti-*P. gingivalis* LPS antibody.

### Comparison of anti-P. gingivalis LPS Ab between RA subsets and controls

Measurement of anti-*P. gingivalis* LPS Ab indicated increased levels in RA patients without CLD, and overall RA patients, compared with HCs (Table 4). Thus, compared with HCs, higher anti-*P. gingivalis* LPS Ab amounts were noted in overall RA cases, especially CLD(−)RA.

**TABLE 4:** A comparison of anti-*P. gingivalis* LPS Ab between RA subsets and controls.

	*t*-test	*U*-test
	*P*	*P*
ILD	0.1320	0.5779
AD	0.2787	0.7351
EMP	0.1066	0.4272
CLD(+)	0.1432	0.7559
CLD(-)	0.0412	0.0061
Overall RA	0.0161	0.1658
	**Anti-*P.gingivalis* Ab, X10^3^U/mL (SD)**	
Overall RA	259.5 (1793.3)	
HC (*n* = 52)	77.6 (183.7)	

RA, rheumatoid arthritis; ILD, interstitial lung disease; AD, airway disease; EMP, emphysema; CLD, chronic lung disease; HCs, healthy controls; SD, standard deviation; LPS, lipopolysaccharide; Ab, antibody. Differences compared with the HC group were tested with the Student’s *t*-test or Mann–Whitney *U*-test. SDs are shown in parentheses.

## Discussion

In this study, it was revealed that anti-*P. gingivalis* LPS Ab amounts reported for RA with EMP were lower compared with RA without CLD and this association was independent. Additionally, higher anti-*P. gingivalis* LPS Ab amounts were observed for RA cases without CLD compared with HCs. In RA without CLD, citrullinated peptides are mainly generated in the oral cavity under the influence of periodontal disease, leading to the production of ACPA. In RA with CLD, especially with EMP, citrullinated peptides are mainly generated in the lung under the influence of smoking leading to the production of ACPA. These findings indicate the pathogenesis of RA might be heterogeneous. It was reported that the endogenous peptidyl-arginine-deiminase of *P. gingivalis* develops citrullinated peptides as autoantigens of ILD, leading to the production of ACPA (30). However, our data did not support that hypothesis. RA without CLD, “oral cavity RA”, would be partially overlapped with younger age onset RA populations and RA patients with CLD, “lung RA”, would be partially overlapped with elder age onset RA populations. The precise information of these heterogeneous features of RA should be investigated and would help to establish personalized medicine of RA.

In previous studies, increased amounts of anti-*P. gingivalis* Ab were reported for RA cases compared to HCs (10). We confirmed increased amounts of anti-*P. gingivalis* Ab in RA cases, *per se*, and they were extremely elevated in the CLD(−)RA group. Because few studies of anti-*P. gingivalis* Ab amounts in RA with CLD have been reported, our study results could not be precisely compared with previous reports. Amounts of anti-*P. gingivalis* Ab in cases with EMP or bronchitis were reported to be comparable (31). However, significantly lower amounts of anti-*P. gingivalis* Ab were reported for RA with EMP than for RA without CLD in this study. Because a similar pathogenesis is suspected to be involved in periodontal disease and EMP (32), these two disorders might have a similar role in RA pathogenesis in patients without CLD or with EMP, respectively.

Amounts of anti-*P. gingivalis* Ab in RA might be influenced by clinical manifestations. However, the relationship between anti-*P. gingivalis* Ab levels and EMP remained significant, even after clinical manifestation effects were adjusted for multiple logistic regression analyses. Thus, the amount of anti-*P. gingivalis* Ab measured was independently linked to EMP in RA.

It was known that ILD and AD resulted in a poor prognosis in RA (3–7). The coexistence of EMP and ILD conferred a poorer prognosis in RA (8). However, few studies on the prognosis of RA patients with EMP were conducted. Thus, the precise characterization of the RA subpopulation with EMP should be performed.

This study reports previously unknown findings related to anti-*P. gingivalis* Ab amounts in RA cases with EMP including low levels in RA complicated by EMP. However, anti-*P. gingivalis* Ab amounts in RA with other CLD including AD and ILD were lower than CLD(−)RA, when examined with *U*-test. The stratified analyses of anti-*P. gingivalis* LPS Ab or ACPA titers on smoking status were conducted, and anti-*P. gingivalis* LPS Ab titers in RA with EMP were lower than CLD(−)RA in never smoker group. However, anti-*P. gingivalis* Ab amounts in RA with other CLD including AD and ILD were lower than CLD(−)RA, when examined with *U*-test. ACPA titers in ever smoker group were higher than those in never smoker group in CLD(−)RA, when examined with *U*-test. The correlations of anti-*P. gingivalis* LPS Ab titers with ACPA or DAS28 were also analyzed and the correlation of anti-*P. gingivalis* LPS Ab titers and DAS28 was found in the RA patients. Since considerable variations of anti-*P. gingivalis* LPS Ab titers between individuals were detected in this study and sample size of the present study was modest, statistical powers were not enough for these subgroup analyses. Study limitations included the modest sample size and the fact that only Japanese populations were included. Thus, large-scale studies of different ethnic populations should be undertaken. Information on periodontal disease reported for patients with RA was not attained here; thus, putative direct correlations between periodontal disease and EMP in RA patients require further examination.

## Conclusion

We investigated anti-*P. gingivalis* LPS Ab in RA with or without CLD. Anti-*P. gingivalis* LPS Ab levels in RA with EMP were lower compared with RA without CLD. Multiple logistic regression analyses showed the independence of this association. Anti-*P. gingivalis* LPS Ab amounts were increased in RA without CLD compared with healthy controls. Citrullinated peptides were mainly generated in the lung of RA with CLD, “lung RA”, and in the oral cavity of RA without CLD, “oral cavity RA”. RA without CLD would be partially overlapped with younger age onset RA populations and RA patients with CLD would be partially overlapped with elder age onset RA populations. These data suggested the heterogeneity of RA and the precise information of these heterogeneous features of RA would help to establish personalized medicine of RA.

## Data Availability

The original contributions presented in this study are included in this article/Supplementary material, further inquiries can be directed to the corresponding author.
