# Supplementary material for: Anti-Porphyromonas gingivalis lipopolysaccharide antibody in rheumatoid arthritis patients with emphysema
Source: Front Med (Lausanne). 2025 Sep 22;12:1654271. doi: 10.3389/fmed.2025.1654271 (PMC12497748; doi:10.3389/fmed.2025.1654271)
Supplement: Supplementary file 1 [file Data_Sheet_1.pdf]

Supplementary Table S1. Correlations of anti-*P. gingivalis* Ab, ACPA, and DAS28 in the RA patients.

Anti−P. gingivalisAb vs. ACPA	Correlation coefficient (95%CI)	*P*
ILD	−0.02 (−0.17−0.14)	0.8153
AD	0.07 (−0.08−0.22)	0.3777
EMP	−0.14 (−0.44−0.18)	0.3883
CLD(+)	0.02 (−0.09−0.12)	0.7601
CLD(−)	0.03 (−0.09−0.15)	0.6539
Overall RA	0.02 (−0.06−0.10)	0.6733
**Anti−P. gingivalisAb vs. DAS28**	**Correlation coefficient (95%CI)**	***P***
ILD	0.20 (0.00−0.38)	0.0453
AD	−0.05 (−0.22−0.12)	0.5369
EMP	0.03 (−0.30−0.35)	0.8669
CLD(+)	0.00 (−0.12−0.12)	0.9940
CLD(−)	0.14 (0.02−0.26)	0.0225
Overall RA	0.09 (0.00−0.17)	0.0470
**ACPA vs. DAS28**	**Correlation coefficient (95%CI)**	***P***
ILD	−0.04 (−0.24−0.16)	0.6870
AD	0.04 (−0.13−0.21)	0.6351
EMP	−0.26 (−0.54−0.08)	0.1300
CLD(+)	−0.04 (−0.16−0.08)	0.5367
CLD(−)	0.09 (−0.03−0.21)	0.1460
Overall RA	0.03 (−0.06−0.12)	0.4919

RA: rheumatoid arthritis, ILD: interstitial lung disease, AD: airway disease, EMP: emphysema, CLD: chronic lung disease, ACPA: Anti-citrullinated peptide antibody, DAS: disease activity score, CI: confidence interval. Pearson correlation coefficient value and 95%CI of each group was shown. Correlation coefficients between anti-*P. gingivalis* Ab, ACPA, and DAS28 were calculated.
