# Supplementary material for: Anti-Porphyromonas gingivalis lipopolysaccharide antibody in rheumatoid arthritis patients with emphysema
Source: Front Med (Lausanne). 2025 Sep 22;12:1654271. doi: 10.3389/fmed.2025.1654271 (PMC12497748; doi:10.3389/fmed.2025.1654271)
Supplement: Supplementary file 2 [file Data_Sheet_2.pdf]

Supplementary Table S2. Anti-*P. gingivalis* LPS Ab in the RA patients with or without smoking habit.

		Comparison with CLD(−) ever smoker			Comparison with CLD(−) never smoker		Comparison between ever and never smoker	
	Ever smoker	t-test	U-test	Never smoker	t-test	U-test	t-test	U-test
	Anti-*P.gingivalis* Ab, X10^3U/mL (SD)	*P*	*P*	Anti-*P.gingivalis* Ab, X10^3U/mL (SD)	*P*	*P*	*P*	*P*
ILD	164.8 (681.1)	0.2669	0.1888	141.6 (542.1)	0.1326	$7.38X10^{-5}$	0.8170	0.0636
AD	96.2 (382.6)	0.2101	0.0088	268.9 (1645.8)	0.9515	0.0063	0.3348	0.3050
EMP	37.8 (72.3)	0.1694	0.0173	18.9 (16.4)	0.0003	0.0928	0.2200	0.5877
CLD(+)	116.2 (513.7)	0.2239	0.0093	203.3 (1231.5)	0.5049	$3.07X10^{-5}$	0.3852	0.6820
CLD(-)	728.9 (4455.8)			280.2 (911.0)			0.3751	0.5668
Overall RA	323.9 (2633.1)			240.2 (1088.6)			0.6441	0.4349

RA: rheumatoid arthritis, ILD: interstitial lung disease, AD: airway disease, EMP: emphysema, CLD: chronic lung disease, SD: standard deviation, LPS: lipopolysaccharide, Ab: antibody. The mean of each group is shown. SDs are shown in parentheses. Differences compared with the CLD(-) population were tested with the Student's t-test or Mann-Whitney U-test. Differences compared between ever and never smoker were also tested with the Student's t-test or Mann-Whitney U-test and are shown in the right column.
