# Supplementary material for: Anti-Porphyromonas gingivalis lipopolysaccharide antibody in rheumatoid arthritis patients with emphysema
Source: Front Med (Lausanne). 2025 Sep 22;12:1654271. doi: 10.3389/fmed.2025.1654271 (PMC12497748; doi:10.3389/fmed.2025.1654271)
Supplement: Supplementary file 3 [file Data_Sheet_3.pdf]

Supplementary Table S3. ACPA in the RA patients with or without smoking habit.

		Comparison with CLD(−) ever smoker			Comparison with CLD(−) never smoker		Comparison between ever and never smoker	
	Ever smoker	t−test	U−test	Never smoker	t−test	U−test	t−test	U−test
	ACPA, U/ml (SD)	*P*	*P*	ACPA, U/ml (SD)	*P*	*P*	*P*	*P*
ILD	254.9 (248.4)	0.1416	0.2741	404.3 (888.6)	0.1116	0.0591	0.1482	0.4376
AD	239.6 (285.2)	0.1120	0.0970	287.0 (377.6)	0.3282	0.2672	0.3897	0.4750
EMP	455.5 (407.1)	0.1242	0.0635	415.4 (349.3)	0.3321	0.2367	0.8244	0.9248
CLD(+)	288.3 (306.9)	0.4091	0.4372	344.0 (660.9)	0.0631	0.0569	0.3129	0.8884
CLD(−)	325.3 (326.5)			242.3 (296.4)			0.0608	0.0217
Overall RA	300.6 (313.3)			296.4 (522.2)			0.8919	0.1309

RA: rheumatoid arthritis, ILD: interstitial lung disease, AD: airway disease, EMP: emphysema, CLD: chronic lung disease, SD: standard deviation, ACPA: anti−citrullinated peptide antibody. The mean of each group is shown. SDs are shown in parentheses. Differences compared with the CLD(−) population were tested with the Student’s t−test or Mann−Whitney U−test. Differences compared between ever and never smoker were also tested with the Student’s t−test or Mann−Whitney U−test and are shown in the right column.
